# Enhancing Bird-Strike Resistance of Aircraft Canopies via Nanoparticles: A Strain-Rate-Dependent Micromechanical (SRDM) and Numerical Approach

**DOI:** 10.3390/polym18121439

**Published:** 2026-06-09

**Authors:** Ferhat Demir, Ugur Simsek, Mesut Kirca

**Affiliations:** 1 Department of Mechanical Engineering, Istanbul Technical University, 34437 Istanbul, Türkiye; demirferh@itu.edu.tr; 2 Department of Mechanical Engineering, Ozyegin University, 34794 Istanbul, Türkiye; 3 Mechanical and Aerospace Systems Research Group, Faculty of Engineering, University of Nottingham, Nottingham NG7 2TU, UK; ugur.simsek@nottingham.ac.uk

**Keywords:** polymer, nanocomposite, micromechanics, transparency, canopy, bird strike

## Abstract

Aerospace canopies require both high impact resistance and optical transparency for pilot safety and aerodynamic shielding. While polycarbonate (PC) and poly(methyl methacrylate) (PMMA) are widely utilized, their vulnerability to strain-rate-dependent failure during high-velocity bird strikes necessitates advanced reinforcement strategies. This study presents a multiscale computational framework for nanoparticle-reinforced PC nanocomposites. To circumvent the prohibitive computational costs of atomistic simulations, a novel Strain-Rate Dependent Micromechanics (SRDM) framework is proposed for silica-, alumina-, and zirconia-reinforced PC systems, integrating the Goldberg constitutive model with Halpin–Tsai micromechanics to generate rate-dependent stress–strain responses and calibrate Johnson–Cook (J-C) parameters for impact-scale simulations. Unlike conventional approaches relying on atomistic simulations or empirical fitting, the proposed framework directly links micromechanical nanocomposite modeling with finite element bird-strike simulations. Bird-strike analyses were performed in LS-DYNA on a generic fighter canopy model. The framework further incorporates literature-based optical transparency criteria considering nanoparticle size and refractive-index compatibility. Among the investigated nanofillers, silica-reinforced PC provided the most favorable response. At the most critical impact location, the maximum canopy deformation decreased from 118.6 mm for neat PC to 61.9 mm, corresponding to an approximately 48% reduction. Although the reinforced canopy exhibited a reduction in peak internal energy absorption from approximately 10 kJ to 5 kJ due to its increased stiffness and reduced plastic deformation, it provided improved deformation resistance and structural stability under impact loading. Overall, this work provides a computationally efficient framework for designing bird-strike-resistant transparent nanocomposite canopy structures using nanofiller systems previously reported in the literature to preserve optical transparency.

## 1. Introduction

Bird strikes pose a critical threat to aviation safety, capable of inflicting severe damage on aircraft structures and endangering personnel [1]. Due to impact forces that scale with the square of velocity, forward-facing transparency components are particularly susceptible to failure [2,3]. Studies indicate that canopies are involved in over 21% of reported strikes, highlighting their vulnerability despite their essential role in pilot protection [4]. This hazard is particularly acute for military aircraft utilizing low-altitude, high-speed flight envelopes where bird populations are dense [5]. However, across all aviation sectors, the highest frequency of incidents is consistently recorded during take-off and landing in the vicinity of airfields [6].

To mitigate the catastrophic consequences of such impacts, modern military canopies have transitioned from traditional brittle glass to advanced ductile materials, primarily polycarbonate (PC) and poly(methyl methacrylate) (PMMA) [7]. Beyond their fundamental roles in maintaining cabin pressure and pilot visibility, these structures must adhere to strict airworthiness standards, including the requirement to sustain a 4 lb (1.8 kg) bird impact at cruise velocity without structural breach [8]. This requirement presents a significant materials engineering challenge, as high-velocity strikes generate intense impulse loads and kinetic energy transfer [9]. While composite configurations utilizing alternating layers of PC and PMMA are currently used to improve ballistic performance in armor systems, their constitutive behavior at high strain rates remains insufficiently characterized [10]. Moreover, although these polymers provide superior specific strength, optical clarity, and formability [11], their performance is often compromised under extreme dynamic conditions, where they may exhibit brittle fracture characteristics and inadequate energy dissipation despite their inherent quasi-static toughness [12]. Recent advances in transparent polymer nanocomposites have further demonstrated that high optical transparency and improved mechanical performance can be achieved simultaneously through controlled nanoparticle dispersion and interface engineering [13].

To overcome the intrinsic limitations of conventional monolithic materials, significant research effort has been directed toward polymer-based nanocomposites. Jordan et al. [14] examined polymer matrix nanocomposites, noting that nano-sized fillers introduce new phenomena that significantly enhance mechanical properties like modulus and strength compared to conventional composites. Their review emphasizes that proper processing techniques are essential to realize these improvements and address the inherent shortcomings of monolithic polymers. Adhikari [15] highlights that polymer nanoparticles, due to their high surface-to-volume ratio, exhibit superior physical and chemical properties that surpass the limitations of bulk polymer structures. This work illustrates how these nanomaterials enable the development of multi-functional systems for advanced applications. Bedi et al. [16] employed a multiscale simulation approach to demonstrate that carbon nanotube and graphene reinforcements substantially improve the tensile strength and elastic moduli of epoxy systems. Their results prove that integrating nano-reinforcements effectively overcomes the mechanical constraints of pure epoxy resins, significantly enhancing their load-bearing capacity. Consequently, the reinforcement of matrices such as PMMA and PC with nanofillers has emerged as a pivotal area of inquiry, driven by the capacity of these nanostructures to augment energy dissipation, stiffness, and ductility [17,18]. However, characterizing these materials for aerospace applications presents specific difficulties; high-velocity events, such as bird strikes or debris collisions, induce extreme strain rates and complex stress wave propagation that necessitate high-fidelity constitutive models [19,20]. Given that polymer mechanical properties are exceptionally sensitive to both temperature and deformation rate, standard modeling frameworks often fail to accurately predict the transition from ductile to brittle behavior at these elevated speeds [21]. Recent studies have further demonstrated that incorporating strain-rate-dependent constitutive formulations into finite element impact frameworks significantly improves the prediction of polymer composite response under dynamic loading conditions [22,23]. Furthermore, a critical trade-off exists between mechanical reinforcement and optical performance. While nanoparticle integration enhances toughness, it risks compromising transparency through light scattering, a phenomenon governed by refractive index mismatches and particle dimensions; preserving transmittance above the critical 80% threshold generally requires filler sizes smaller than 40 nm [24]. Thus, while such nanocomposites demonstrate improved impact potential, optimizing the equilibrium between enhanced energy dissipation and strict optical clarity requirements remains a significant engineering challenge [25].

Consequently, this study proposes a systematic approach to evaluate novel nanoparticle-reinforced PC composites, targeting improved impact resistance and reduced structural deformation while rigorously defining design criteria to meet the stringent transparency requirements of canopy structures. Since the reliability of impact assessments depends directly on the fidelity of material modeling, a robust methodology is essential to capture multiscale behavior under high-strain-rate conditions [26]. To address this sensitivity and circumvent the prohibitive computational costs associated with atomistic simulations, the Strain-Rate Dependent Micromechanics (SRDM) method is employed as the primary analytical strategy [27]. Similar strain-rate-sensitive constitutive and finite element approaches have also recently been employed for the dynamic assessment and validation of composite structures subjected to impact loading conditions [22]. By integrating the non-linear Goldberg polymer model with micromechanical theories such as the Halpin–Tsai equation, the SRDM approach facilitates the precise, analytical derivation of rate-dependent Johnson–Cook (J-C) parameters, enabling the derivation of rate-dependent constitutive parameters for representing deformation response and impact-energy absorption trends under dynamic loading conditions [28].

A critical variable in this approach is the nanoparticle-to-polymer ratio, which is governed by competing optical and manufacturing constraints. To maintain optical clarity, nanoparticle diameters are generally restricted to below 40 nm, as scattering intensity increases sharply with particle size in accordance with Rayleigh’s law [29]. In addition, transparency is significantly enhanced by minimizing the refractive index mismatch between the matrix and filler [26]; PMMA (≈1.49) and PC (≈1.58) exhibit high compatibility with nanoparticles such as nano-silica (*n* ≈ 1.46), thereby mitigating scattering losses [30,31]. In addition to these optical thresholds, the upper limit of the nanoparticle-to-polymer ratio (wt%) is dictated by manufacturability. Issues such as nanoparticle agglomeration typically restrict feasible reinforcement levels to below 10 wt%; exceeding this range leads to aggregation and turbidity, which compromise both the mechanical homogeneity and the optical transmission of the final component [32,33].

Accordingly, this study addresses the gap between material-level nanocomposite design and structural bird-strike assessment of transparent aircraft canopies. Previous studies have generally treated these aspects separately: nanocomposite micromechanics has mainly been used to estimate material-level reinforcement effects [14,15,16], strain-rate-dependent constitutive models have been developed primarily from coupon-scale experimental data [21,22,26], and canopy- or panel-level bird-strike simulations commonly rely on predefined monolithic polymer properties or experimentally calibrated material parameters [6,7]. In contrast, the present work links these levels within a single screening-oriented computational workflow by deriving strain-rate-dependent nanocomposite stress–strain responses using the SRDM approach, calibrating the corresponding J-C parameters [34], and directly implementing them in LS-DYNA [35] bird-strike simulations of a generic fighter canopy. Therefore, the novelty lies not in the isolated use of micromechanics, J-C plasticity, or explicit impact simulations, but in their synergistic integration to design transparent nanoparticle-reinforced PC canopies under simultaneous optical, manufacturability, and high-rate impact constraints. This framework enables the comparative evaluation of silica-, alumina-, and zirconia-reinforced PC systems and helps narrow the design space before costly experimental development.

## 2. Materials and Methods

**Figure 1:**
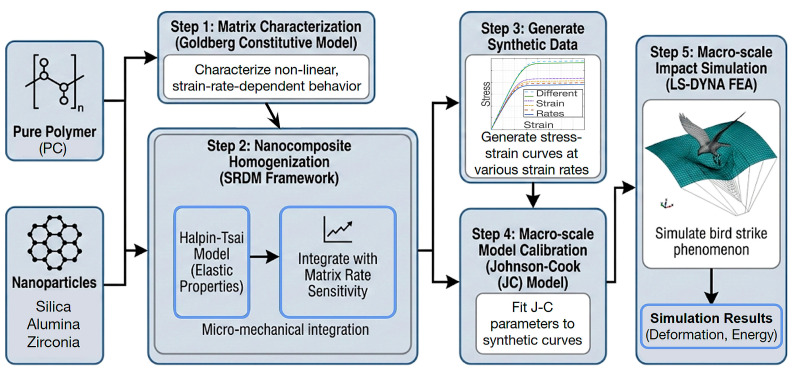
Multiscale computational workflow for nanocomposite impact analysis (SRDM-FEM).

In this study, the Strain-Rate Dependent Micromechanics (SRDM) method was employed to computationally derive the strain-dependent material properties of polymer nanocomposites, specifically PC reinforced with nanoparticles. The performance of these engineered materials was subsequently evaluated under high-velocity impact conditions using Finite Element Analysis (FEA). The overall workflow of the proposed approach is illustrated in Figure 1. The methodology proceeds in four distinct phases: First, the non-linear, strain-rate-dependent behavior of the pure polymer matrix was characterized using the Goldberg constitutive model [36]. Second, the instantaneous matrix properties derived from the Goldberg model were combined with the properties of the nanofillers via the Halpin–Tsai micromechanical equation [37]. This integration defines the SRDM framework, effectively coupling the matrix’s rate sensitivity with the elastic reinforcement provided by the nanostructures. Subsequently, synthetic stress–strain curves were generated for the nanocomposites across a range of strain rates; these curves serve as the calibration dataset for the J-C material model. Finally, the calibrated J-C parameters were implemented into a macro-scale LS-DYNA environment to simulate the bird-strike phenomenon.

### 2.1. Constitutive Modeling of the Polymer Matrix

Polymeric materials are highly sensitive to loading rates; consequently, various constitutive models have been developed to characterize their rate-dependent mechanical behavior [38,39,40,41]. In the present work, the constitutive framework proposed by Goldberg et al. [36] is employed to capture the strain-rate-dependent behavior of the pure polymer matrix. This model modifies standard total strain theory by assuming the total strain ($\varepsilon^{T}$) consists of two distinct components: an elastic term $\left(\varepsilon^{E}\right)$ and an inelastic term $\left(\varepsilon^{I}\right)$. Consequently, within this framework, the inelastic strain rate $\left({\dot{\varepsilon }}_{ij}^{I}\right)$ is defined as follows [36]:

(1) $${\dot{\varepsilon }}_{ij}^{I}=2D_{0}\;exp[-\frac{1}{2}{\left(\frac{Z}{\sigma_{e}}\right)}^{2n}](\frac{S_{ij}}{2\sqrt{J_{2}}}+\alpha \delta_{ij})$$

where the inelastic strain rate (${\dot{\varepsilon }}_{ij}^{I}$) is expressed as a function of the deviatoric stress tensor ($S_{ij}$), the second invariant of the deviatoric stress tensor ($J_{2}$); and the internal state variables Z and α representing isotropic hardening and hydrostatic stress effects, respectively. Additionally, $D_{0}$ and n serve as distinct material constants; $D_{0}$ defines the maximum inelastic strain rate limit; while n governs the rate dependency of the material. To account for pressure sensitivity, the effective stress $\sigma_{e}$ explicitly incorporates the influence of the hydrostatic stress component $\sigma_{kk}$ and the hydrostatic stress coefficient α as follows:

(2) $$\sigma_{e}=\sqrt{{3J}_{2}}+\sqrt{3}\alpha \sigma_{kk}$$

where $\sigma_{kk}$ denotes the sum of the normal stress components (equivalent to three times the mean stress), serving as a state variable that governs the influence of hydrostatic stress. Furthermore, the deviatoric stress $S_{ij}$ is stated as:

(3) $$S_{ij}=\;\sigma_{ij}-\frac{1}{3}\sigma_{kk}\delta_{ij}$$

where $\sigma_{ij}$ is the Cauchy stress tensor of the pure polymer matrix, $\delta_{ij}$ is the Kronecker delta, and $\sigma_{kk}/3$ represents the mean hydrostatic stress. To capture the strain hardening behavior, the evolution of the two internal state variables, Z and α is computed using the following constitutive rate equations:

(4) $$\dot{Z}=q(Z_{1}-Z){\dot{e}}_{e}^{I}$$

(5) $$\dot{\alpha }=q(\alpha_{1}-\alpha ){\dot{e}}_{e}^{I}$$

(6) $${\dot{e}}_{e}^{I}=\sqrt{\frac{2}{3}{\dot{e}}_{ij}^{I}{\dot{e}}_{ij}^{I}}$$

(7) $${\dot{e}}_{ij}^{I}={\dot{\varepsilon }}_{ij}^{I}-\frac{{\dot{\varepsilon }}_{kk}^{I}}{3}\delta_{ij}$$

where q is a material constant representing the hardening rate. The parameters $Z_{1}$ and $\alpha_{1}$ define the maximum limits of Z and α, respectively, while $Z_{0}$ and $\alpha_{0}$ establish their initial values. Additionally, the term ${\dot{e}}_{e}^{I}$ represents the effective deviatoric inelastic strain rate ${\dot{e}}_{ij}^{I}$.

The six Goldberg model parameters (n, $Z_{0}$, $Z_{1}$, q, $\alpha_{0}$, $\alpha_{1}$) are identified by fitting the model predictions to experimental engineering stress–strain data of the target polymer matrix across multiple strain rates, using a weighted nonlinear least-squares algorithm with a sum-of-squared-residuals objective function. The elastic modulus of the polymer matrix E, used to relate the elastic strain to stress within the Goldberg framework, and the reference inelastic strain rate $D_{0}$, treated as a scale factor set equal to 10^6^ s^−1^ following Goldberg et al. [36] and Shokrieh et al. [42], are fixed a priori from the literature and excluded from the optimization.

The introduced constitutive Equation (1) is an implicit equation and it cannot be solved analytically. To ensure the stability and accuracy of the differential equation solutions, a forward Euler numerical integration scheme [43] with an appropriately refined timestep was implemented in the present study. The literature indicates that the Poisson’s ratio of composites is insensitive to strain rate variations [44]. Because this composite property is a direct function of its constituents, it is correspondingly assumed here that the Poisson’s ratios of both the polymer matrix and the fibers are independent of the loading rate.

### 2.2. Halpin–Tsai Micromechanics Model

With the rate-dependent behavior of the pure polymer matrix established via the Goldberg model, the next step is to evaluate how the addition of nanoparticles alters the overall stiffness of the material. To achieve this, the Halpin–Tsai micromechanics model is utilized to calculate the effective elastic constants of the resulting composite material [37]. Although originally developed for macroscopic composites, this model has been extensively validated in the literature for various nanoscale fillers, including 0-D spherical nanoparticles, 1-D nanotubes or nanofibers, and 2-D nanosheets [45,46,47].

It is crucial to note that in practical nanocomposite fabrication, high-aspect-ratio fillers such as nanotubes or nanosheets are rarely perfectly aligned in a single direction; instead, they are randomly dispersed throughout the polymer matrix [48,49]. To accurately capture this isotropic macroscopic behavior arising from randomly oriented anisotropic fillers, the overall effective elastic modulus of the nanocomposite $E_{Rand}$ must be evaluated using orientation averaging models [48,49,50]. In this approach, the longitudinal modulus $E_{11}$ and the transverse modulus $E_{22}$ of the nanocomposite are first calculated independently using the Halpin–Tsai equations:

(8) $$E_{c}=E_{m}(\frac{1+\xi \eta \nu_{f}}{1-\eta \nu_{f}})$$

(9) $$\eta =\frac{\left(E_{f}/E_{m})-1\right.}{\left(E_{f}/E_{m})+\xi \right.}$$

where $E_{m}$ and $E_{f}$ represent the elastic moduli of the polymer matrix and the nanofiller, respectively, while $\nu_{f}$ is the filler volume fraction. For the longitudinal modulus $E_{11}$, the shape factor ξ is dependent on the filler’s aspect ratio; it is formulated as for tubular, fibrillar, or whisker-like nanoparticles where l and d represent the filler length and diameter, respectively, for nanosheets where t is the thickness and ξ=2 for isotropic spherical fillers [51,52]. Conversely, for the transverse modulus $E_{22}$, the shape factor ξ is approximated as ξ=2 [48].

Subsequently, if the nanoparticles are assumed to have a completely random spatial orientation in three dimensions (3D random distribution), the effective isotropic modulus of the nanocomposite is calculated using the established averaging equation [48,49]:

(10) $$E_{Rand-3D}=\frac{1}{5}E_{11}+\frac{4}{5}E_{22}$$

Alternatively, if the manufacturing process (such as thin-film casting) restricts the nanoparticles to a two-dimensional in-plane random orientation (2D random distribution), the effective modulus is approximated using the Halpin–Pagano laminate analogy [50,51].

(11) $$E_{Rand-2D}=\frac{3}{8}E_{11}+\frac{5}{8}E_{22}$$

It should be noted that the present micromechanical framework assumes uniform nanoparticle dispersion, ideal interfacial bonding, and homogeneous distribution within the polymer matrix. Potential effects associated with nanoparticle agglomeration, imperfect interfacial adhesion, and local manufacturing-induced heterogeneities are therefore not explicitly captured within the current Halpin–Tsai-based homogenization approach.

In the present SRDM framework, the matrix modulus $E_{m}$ in Equation (8) is not treated as a constant but is substituted at each strain increment by the instantaneous tangent modulus computed from the Goldberg-predicted stress–strain curve of the neat polymer. The resulting strain-dependent composite modulus is then integrated incrementally over the strain history to construct the full synthetic stress–strain curves of the nanocomposite across all strain rates. All nanofiller properties, including elastic modulus, density, and aspect ratio, are sourced directly from the literature and are not subject to fitting.

### 2.3. Macro-Scale Model Calibration

While the micromechanical approach successfully establishes the effective elastic constants of the reinforced polymer, simulating high-velocity impacts requires a robust definition of the material’s macroscopic flow stress and plastic deformation across varying strain rates. To address this, the empirical Johnson–Cook (J-C) constitutive model is employed to characterize the dynamic response of the nanocomposite. Although the J-C model was originally developed for metallic materials, previous studies have successfully applied J-C-type formulations to polycarbonate and polymer-based materials under high-strain-rate impact loading conditions [21,27,53]. Nevertheless, the simplified J-C formulation does not explicitly capture time-dependent viscoelastic relaxation mechanisms characteristic of polymers. The J-C model defines the equivalent von Mises flow stress as a function of strain, strain rate, and temperature [34]. However, in the present study, thermal effects are neglected to focus on the strain-rate-dependent mechanical response of the nanocomposite, following the approach proposed by Shokrieh et al. [53]. In addition, all simulations were performed under isothermal conditions at room temperature (T = 293 K). Accordingly, the J-C model is expressed in its simplified, temperature-independent form as:

(12) $$\sigma =[A+B{\left(\varepsilon_{p}\right)}^{n}][1+C\ln\left({\dot{\varepsilon }}^{*}\right)]$$

In this formulation, A, B, C, and n are the material constants of the temperature-independent J-C model. The parameter A corresponds to the yield stress at the reference strain rate. The constants B and n define the strain hardening behavior, representing the hardening coefficient and exponent, respectively. The parameter C characterizes the strain rate sensitivity. Here, σ denotes the flow stress, $\varepsilon_{p}$ is the equivalent plastic strain, and ${\dot{\varepsilon }}^{*}$ is the dimensionless plastic strain rate, defined as the ratio of the plastic strain rate $\dot{\varepsilon }$ to the reference strain rate $\dot{\varepsilon_{0}}$:

(13) $${\dot{\varepsilon }}^{*}=\frac{\dot{\varepsilon }}{\dot{\varepsilon_{0}}}$$

The strain and strain rate are considered two independent phenomena and can therefore be treated separately. Accordingly, their combined effect on the flow stress is obtained by multiplying the corresponding terms. This independence is exploited to determine the material constants of the J-C model. The parameter A is determined directly from the yield stress obtained from the quasi-static curve. The engineering stress and strain data is subsequently converted into true stress and true strain using Equations (14) and (15), respectively, to ensure an accurate representation of the material behavior in the plastic regime.

(14) $$\sigma_{True}=\sigma_{eng}[1+\varepsilon_{eng}]$$

(15) $$\varepsilon_{True}=ln(1+\varepsilon_{eng})$$

The plastic strain is obtained by subtracting the elastic strain component from the total true strain, as expressed in Equation (16).

(16) $$\varepsilon_{p}=\varepsilon_{True}-\frac{\sigma_{True}}{E}$$

where $\varepsilon_{p}$ denotes the plastic strain, $\varepsilon_{True}$ is the total true strain, $\sigma_{True}$ represents the true stress, and *E* is the elastic modulus of the material. By rearranging Equation (12), the strain hardening relationship can be expressed in logarithmic form as:

(17) $$\log\left(\sigma -A\right)=\log\left(B\right)+n\log\left(\varepsilon_{p}\right)$$

The linearized form enables the determination of the hardening parameters B and n from a linear regression of $\log\left(\sigma -A\right)$ versus $\log\left(\varepsilon_{p}\right)$. In this representation, the slope of the fitted straight line corresponds to the strain hardening exponent n, while the intercept provides $\log\left(B\right)$, from which the hardening coefficient B is obtained. The strain-rate sensitivity parameter C is determined from the slope of the relationship between the normalized dynamic-to-static stress ratio at 2% plastic strain and the natural logarithm of the strain rate [27]. The effective composite elastic modulus computed in Section 2.2 is passed directly to LS-DYNA as the initial elastic modulus of the nanocomposite material card, without further fitting.

### 2.4. Macro-Scale Impact Simulation Approach

**Figure 2:**
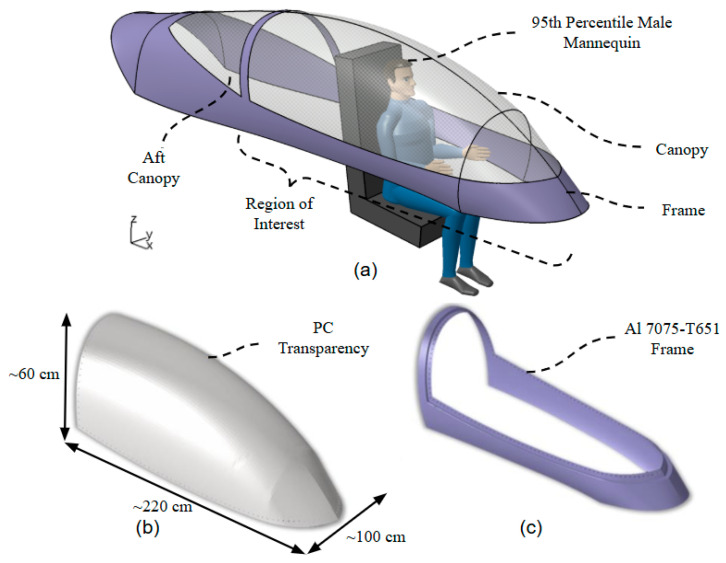
Geometric modelling of a generic fighter canopy system: (**a**) the full assembly including a 95th percentile male mannequin and highlighting the area of interest, and (**b**) the forward PC transparency, and (**c**) the Al 7075-T651 support frame.

Bird-strike simulations are conducted using the nonlinear explicit finite element solver LS-DYNA [35], which is well suited to short-duration, high-rate impact problems characterized by severe nonlinearity and large deformations. For this purpose, first, a representative geometry of a generic fighter aircraft canopy was constructed in CATIA V5-6R2022 [54], as illustrated in Figure 2. The canopy geometry was defined in accordance with established aerodynamic and pilot field-of-view requirements adopted in contemporary military aircraft design [55,56]. A 95th percentile male Digital Human Model generated in CATIA V5 based on Anthropometric Survey data [57] was used to define the internal clearance envelope in compliance with MIL-STD-1472 [58] crew accommodation criteria. The canopy assembly was primarily composed of two structural entities: the transparency (measuring approximately 220 cm in length, 100 cm in width, and 60 cm in height), which acts as the optical and impact-resistant barrier, and the frame, which provides structural rigidity and facilitates attachment to the fuselage. Based on the reported canopy configuration of the F-16 aircraft [59] in the literature, a thickness of 0.75 in (19.05 mm) was adopted for the PC transparency in the present study.

**Figure 3:**
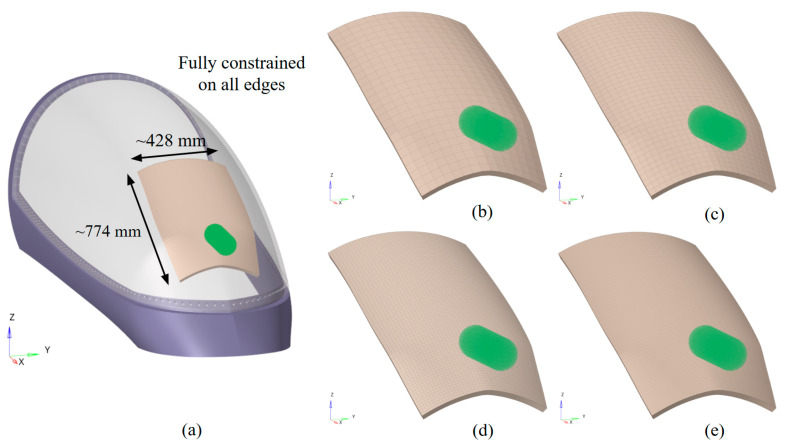
Representative mesh-convergence study performed on the extracted canopy submodel at impact Position 2 (P2): (**a**) location and dimensions of the extracted submodel together with the applied boundary conditions, and representative mesh discretizations with average element sizes of (**b**) 30 mm, (**c**) 20 mm, (**d**) 10 mm, and (**e**) 5 mm.

The geometric model was subsequently imported into HyperMesh 2021 [60] for mesh generation, where a hybrid discretization strategy was employed to accurately capture the distinct mechanical behaviors of the components under high-velocity impact. To determine an appropriate mesh size balancing numerical accuracy and computational efficiency, a mesh-convergence study was conducted using a representative submodel extracted from the central region near impact Position 2 (P2) of the canopy, preserving the local curvature, thickness, material model, boundary conditions, and loading characteristics. Average solid-element sizes of 5, 10, 15, 20, and 30 mm were evaluated, while the maximum out-of-plane displacement at the impact center was monitored as the convergence metric. Coarser meshes significantly underestimated the local deformation response, whereas the difference between the 10 mm and 5 mm discretizations was limited to 2.1 mm (~2.5%), indicating sufficient convergence for the present study. The representative submodel geometry and mesh discretizations are illustrated in Figure 3, while the corresponding convergence results are summarized in Table 1.

In addition, the 19.05 mm thick canopy transparency was consistently discretized using six solid elements through the thickness for all mesh configurations, exceeding the commonly recommended minimum of three-to-four elements for bending-dominated solid analyses [61] and ensuring adequate resolution of through-thickness stress-wave propagation and deformation behavior. Accordingly, an average in-plane element size of 10 mm together with six solid elements through the thickness was adopted for the subsequent full-canopy simulations, providing an acceptable compromise between numerical accuracy and computational cost.

To assess the sensitivity of the explicit dynamic solution to the selected stable time increment, additional simulations were performed using a reduced LS-DYNA time-step scale factor (TSSFAC = 0.667), while the baseline simulations employed the default stable value of TSSFAC = 0.90 recommended in the LS-DYNA Keyword User’s Manual for general explicit analyses [35]. Comparable deformation histories and impact-response characteristics were obtained in both cases, indicating that the numerical solution was insensitive to the selected time-step scaling.

**Figure 4:**
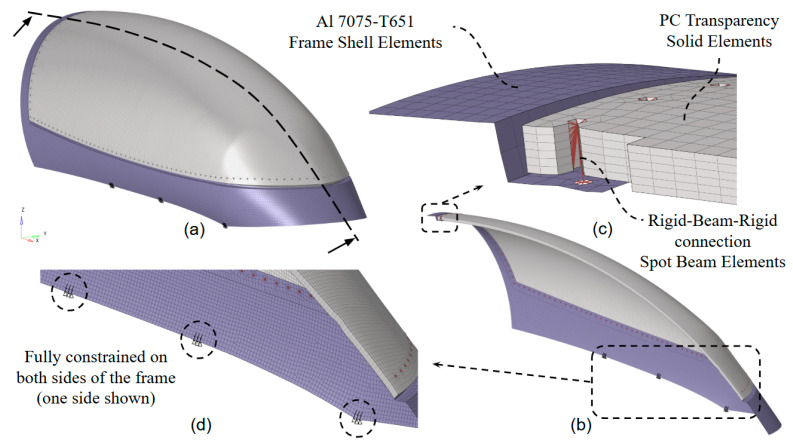
Finite element model of the canopy assembly: (**a**) full model, (**b**) section view, (**c**) fastener modelling using rigid-beam (spot beam) elements, and (**d**) boundary conditions applied at the frame supports (one side shown).

The monolithic transparency was discretized using 186,992 three-dimensional solid elements (SECTION_SOLID) defined by 219,240 nodes, utilizing the constant stress solid element formulation (ELFORM = 1) to effectively resolve through-thickness stress wave propagation. Conversely, the frame structure, characterized by its thin-walled geometry and aluminum alloy composition (Al 7075-T651), was meshed using 17,336 fully integrated shell elements (ELFORM = 16) with 18,035 nodes to mitigate hourglass energy accumulation. The standard mechanical properties for this high-strength aerospace aluminum alloy, specifically, a density of 2.81 g/cm^3^, an elastic modulus of 71.0 GPa, and a Poisson’s ratio of 0.33, were implemented [62]. To accurately predict the elastoplastic deformation and stress distribution under impact loading, the frame was modeled utilizing a piecewise linear plasticity constitutive model (MAT_024_PIECEWISE_LINEAR_PLASTICITY). Figure 4 illustrates the finite element model of the canopy assembly showing the solid-element discretization of the PC transparency and the shell-element discretization of the Al 7075-T651 frame, including representative local mesh details.

The mechanical integration between the transparency and the frame was modeled using a Rigid-Beam-Rigid connection method; specifically, 7.92 mm diameter titanium fasteners were simulated using spotweld beam elements (ELFORM = 9, MAT_100_SPOTWELD). These fasteners were assigned the material characteristics of standard Ti-6Al-4V aerospace bolts, including a density of 4.43 g/cm^3^, an elastic modulus of 114.0 GPa, and a Poisson’s ratio of 0.33 [62]. These beam elements were coupled to the structural mesh via constrained nodal rigid bodies (spider connections) to ensure realistic load transfer. Furthermore, to accurately reflect the airframe mounting conditions, the nodes at six discrete mounting locations, which are symmetrically distributed on both sides of the frame as depicted in Figure 4, were fully constrained against all translational and rotational degrees of freedom using the BOUNDARY_SPC_SET card, thereby establishing a fixed boundary condition.

The strain-rate-dependent mechanical behavior of the PC was modeled using the Simplified J-C constitutive model (MAT_098_SIMPLIFIED_JOHNSON_COOK). For the baseline configuration, the quasi-static mechanical constants, a density of 1.20 g/cm^3^, an elastic modulus of 2.56 GPa, a Poisson’s ratio of 0.39, and an initial yield stress of 55 MPa, were directly adopted from the experimental findings of Sarıkaya et al. [21]. For the nanocomposite models, these parameters were subsequently updated based on the parameters derived from the SRDM approach. Because the primary objective of this macroscopic structural analysis was to evaluate the extreme dynamic deflection and the energy dissipation mechanisms of the nanocomposite canopy rather than predicting catastrophic rupture, the plastic strain at failure was deliberately set to zero (PSFAIL = 0.0) [35]. This approach prevents macroscopic element deletion, allowing the solver to maintain full structural continuity while focusing on the plastic deformation and energy absorption capabilities.

Given the extreme dynamic pressures generated during high-velocity bird strikes, the volumetric response and shock wave propagation within the PC transparency must be governed by an Equation of State (EoS). In this study, the Grüneisen Equation of State (EOS_GRUNEISEN) was employed. The EoS parameters were defined with a bulk sound speed of 1933 m/s, a linear Hugoniot slope coefficient of 2.65, and a Grüneisen gamma of 0.61 [21].

The bird was modeled as a cylinder with hemispherical ends with a mass of 4 lb (~1.81 kg), a geometry and weight commonly used in bird-strike studies to match real impact characteristics [63]. Its dynamic response and validation will be presented in a later section. In accordance with military aircraft structural design guidelines for low-altitude tactical operations, the relative bird impact velocity was established at 460 knots (~236 m/s) at four designated critical positions [3,55,59]. Under these high-velocity boundary conditions, the analysis was carried out for a duration of 10 ms to capture the complete fluidic squash phase of the bird and the maximum dynamic deflection of the transparency, consistent with established SPH methodologies applied to military aircraft canopy and windshield structures [64,65].

The contact interaction between the SPH bird model and the polymer canopy structure was implemented using the automatic node-to-surface contact algorithm [66]. Following the experimental and numerical observations reported by Shmotin et al. [67], the static and dynamic friction coefficients were both set to zero, resulting in a frictionless contact assumption. Their sensitivity investigation showed that friction had no significant direct influence on the structural strain response, while the frictionless case provided the best agreement with experimental bird-strike data. Therefore, the frictionless assumption was adopted in the present study to represent the predominantly hydrodynamic interaction behavior during impact. Nevertheless, for oblique or grazing impacts, non-zero tangential friction may still influence SPH particle spreading and local deformation patterns.

**Figure 5:**
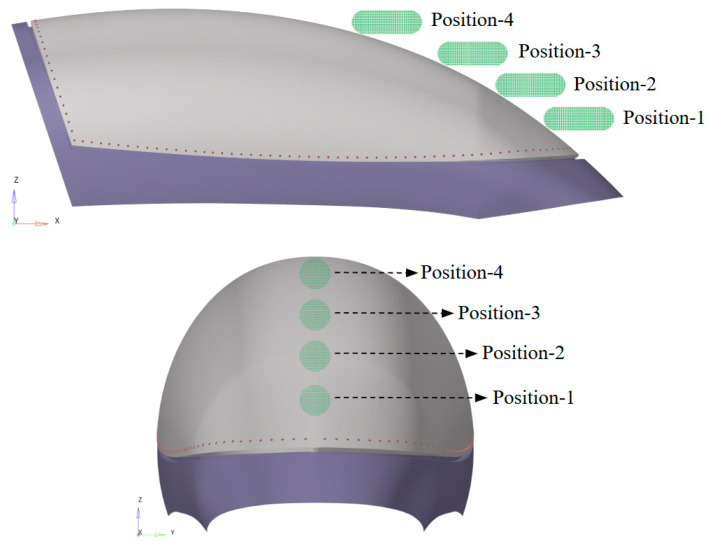
Side and front views of the canopy showing P1–P4 for impact analysis.

To ensure numerical stability and prevent non-physical zero-energy deformation modes during this highly dynamic event, a Flanagan–Belytschko stiffness form hourglass control (IHQ = 4) with a coefficient of 0.03 was applied globally [35]. Furthermore, the explicit time integration was executed with a time-step scale factor (TSSFAC) of 0.90, strictly without the introduction of artificial mass scaling (DT2MS = 0.0), ensuring that the dynamic inertial effects remained purely physical [35]. To comprehensively assess the structural response and potential damage, the validated SPH bird model was impacted at four designated critical positions (P1 to P4) on the canopy as depicted in Figure 5, representing structurally sensitive regions associated with pilot head clearance and Head-Up Display (HUD) integration [68,69].

### 2.5. Selection of Nanoscale Reinforcements

The selection of reinforcing nanofillers for the aircraft canopy was governed by the critical need to minimize deformation and maintain structural integrity under dynamic impact while preserving strict optical transparency. Neat PC, widely utilized in aerospace transparencies, exhibits a baseline visible light transmittance of up to 89%, approaching that of inorganic glass [70]. However, the extreme structural thickness required for bird-strike resistance inherently attenuates this value. Consequently, military and operational aerospace standards establish a strict lower threshold, requiring the final thickened canopy system to maintain a minimum visible light transmittance of approximately 80% in the primary viewing zones [71]. This critical optical boundary acts as the primary design constraint, directly dictating the maximum permissible concentration (weight percentage) of the nanoparticle reinforcements.

To effectively address the competing requirements of bird-strike resistance and optical performance, silica (SiO_2_), alumina (Al_2_O_3_), and zirconia (ZrO_2_) were selected based on existing studies to reinforce the PC matrix. According to Rayleigh scattering theory, maintaining high optical clarity requires dispersed nanoparticles to be smaller than one-tenth of the wavelength of visible light; thus, a critical size threshold of <40 nm was established [24,29]. Furthermore, minimizing the refractive index mismatch between the fillers and the matrix is essential to reduce haze [72,73]. Silica offers excellent refractive index compatibility (*n* ≈ 1.46) with PC (*n* ≈ 1.58), minimizing optical scattering and enabling the use of higher weight fractions beyond 10% [74,75]. However, the inherent hydrophilicity of silica induces severe nanoparticle agglomeration, hindering uniform dispersion within the polymer matrix [76]. To resolve this and maximize material performance, surface modification techniques such as physical adsorption, covalent bonding, or polymer grafting are essential [76,77]. While the selected nanofillers satisfy the minimum visible light transmittance requirement of 80%, optical quality is further ensured by minimizing haze, which arises from scattering due to particle size and refractive index mismatch. This distinction clarifies that both transmittance and clarity are considered in the material selection for the canopy. Conversely, alumina provides superior ballistic protection and kinetic energy dissipation, though its higher refractive index (*n* ≈ 1.76) mandates strict adherence to the lower nanoscale limits to prevent severe optical degradation via Rayleigh scattering [31].

Similarly, zirconia stands out as a formidable reinforcement candidate owing to its outstanding chemical inertness, excellent thermal stability, and exceptional hardness [78]. Despite exhibiting a significantly higher refractive index (*n* ≈ 2.16), the employment of ultra-fine zirconia nanocrystals coupled with targeted surface functionalization effectively suppresses Rayleigh scattering [72,78]. Crucially, for high-velocity impact scenarios such as bird strikes, zirconia is distinguished by its intrinsic high strength and fracture toughness [24]. The integration of these rigid nanoparticles into the polymer matrix not only preserves optical clarity even at remarkably high filler loading fractions but also significantly enhances the energy dissipation capacity and flexural strength of the composite under dynamic loading [79].

Beyond composition, this study also evaluated the mechanical and optical effects of varying nanoparticle morphologies, specifically analyzing 0-D spherical and 1-D fibrillar (nanowhisker) geometries. Notably, although the typical critical size for dispersed nanoparticles is <40 nm to maintain transparency, in previous studies, alumina whiskers with a length of 2800 nm were successfully incorporated into PC while retaining ≥ 80% visible light transmittance [80]. This choice allowed us to directly compare our simulation results with experimental literature data and validate the mechanical improvements obtained using our model. Crucially, the theoretical optical viability of integrating these nanofillers has been corroborated by previous studies, which experimentally validated their high transparency in physical specimens’ fabrication [13,72,81]. All corresponding material, morphological, and transparency properties utilized in this study are comprehensively detailed in Table 2.

The values indicated under ‘Max %wt Allowance’ represent the maximum nanoparticle weight fractions considered for each nanocomposite type. In the present simulations, the actual nanoparticle contents were set to these maximum values for each composite system: 30 wt% for silica, 2 wt% for alumina, and 2.5 wt% for zirconia. These values were selected to maximize reinforcement while remaining within optical and practical manufacturability constraints, ensuring uniform nanoparticle dispersion and feasibility for polymer processing.

## 3. Results

As established in the previous section, the selection of the three specific nanofillers was driven by the concurrent need to preserve the optical transparency of the PC matrix while maximizing its structural reinforcement. Building on these intrinsic material properties, this section provides a comprehensive evaluation of the proposed nanocomposite canopies, systematically linking material-level constitutive modeling with macro-scale dynamic impact behavior.

To validate the approach presented in the previous section, the proposed method is first benchmarked against experimental data from the literature for neat polypropylene (PP) and CNT-reinforced PP nanocomposites [42]. This preliminary step ensures the reliability of the Goldberg model before its application to baseline neat PC, where it demonstrates excellent agreement with experimental stress–strain behavior. To accurately account for the elastic reinforcement provided by the selected nanoparticles, the Halpin–Tsai micromechanical model is incorporated, forming a validated hybrid framework. Using this framework, the SRDM predicts the dynamic stress–strain responses of PC reinforced with the chosen nanofillers across a range of strain rates. These analytically derived curves are then processed through an in-house parameter identification algorithm, which extracts the J-C constitutive parameters and effective elastic moduli for each nanocomposite.

Building on this material-level foundation, the study transitions to the structural scale. The Smoothed Particle Hydrodynamics (SPH) bird model is first validated to ensure accurate replication of high-velocity soft-body impacts. Macro-scale bird-strike simulations are then performed, employing the derived J-C parameters to evaluate the structural performance of the three nanocomposite configurations at four critical canopy locations. For clarity, the discussion emphasizes the most significant improvements in crashworthiness, rather than presenting all displacement profiles and full energy balances (total, kinetic, internal, and hourglass energies from LS-DYNA) across the twelve scenarios. A comprehensive summary highlights the key differences in displacement reduction and impact-energy partitioning relative to the baseline neat PC canopy, establishing a robust benchmark for assessing the trade-off between stiffness and energy absorption in the proposed designs.

### 3.1. Validation of the Proposed SRDM Method

To verify the reliability of the numerical procedure, specifically, the Goldberg constitutive model and the J-C parameter identification routine, before its application to the target PC/oxide nanocomposite systems, a preliminary procedural benchmark was conducted using established literature data for neat PP and CNT-reinforced PP. It is emphasized that this benchmark is intended solely to confirm the computational framework, and does not constitute a validation of the PC-specific material predictions. For this purpose, the dynamic stress–strain response of the PP matrix reinforced with 0.3 wt% CNTs was adopted from the reference study [42].

**Figure 6:**
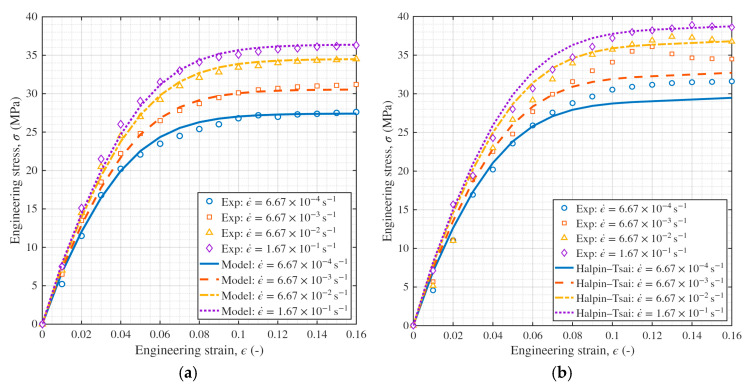
(**a**) Neat PP: experimental data from Shokrieh et al. [42] vs. proposed Goldberg model and (**b**) CNT/PP: experimental data from Shokrieh et al. [42] vs. Halpin–Tsai prediction.

The validation of the in-house-developed MATLAB R2025b tool was performed in successive stages. Initially, the rate-dependent viscoplastic behavior of the neat PP matrix was modelled using the Goldberg constitutive equations. As illustrated in Figure 6a, the algorithmically predicted stress–strain curves for neat PP exhibit a strong correlation with the experimental data across different strain rates. Subsequently, the Halpin–Tsai micromechanical model was integrated to predict the mechanical behavior of the 0.3 wt% CNT/PP nanocomposite. The comparison between the experimental data and the SRDM model predictions for the nanocomposite is presented in Figure 6b, demonstrating the capability of the proposed tool to accurately capture the strengthening effect of the nanoparticles under dynamic loading.

A closer inspection of the results reveals minor discrepancies, particularly at low strain levels where the maximum deviations between the model and experimental points were observed. These localized errors are primarily attributed to the inherent resolution limits of extracting benchmark data from published plots via web-based digitizing applications, a process that is particularly sensitive in the high-curvature transition regions. Furthermore, the slight over-prediction in the early elastic-to-plastic transition can be explained by the idealized nature of the micromechanical models, which do not fully account for the complex viscoelastic lag and potential microstructural irregularities, such as localized CNT agglomeration, that may influence the material’s initial response. Conversely, the slight under-prediction observed at lower strain rates is attributable to the inherent limitations of the Goldberg model in fully capturing the enhanced viscoelastic contributions that become more pronounced at quasi-static loading rates, where molecular chain relaxation mechanisms operate over longer time scales than those to which the model parameters were calibrated.

**Figure 7:**
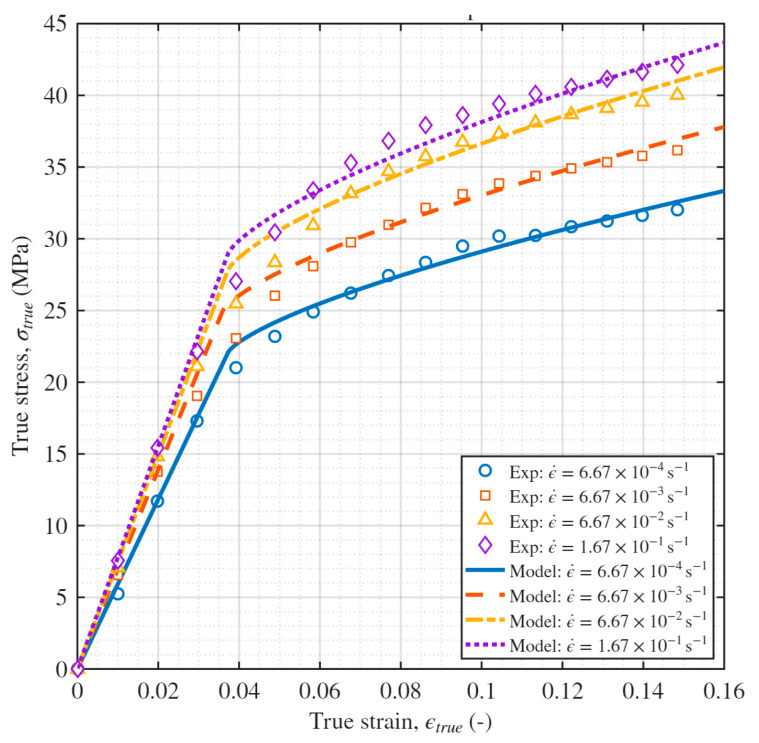
Comparison of the J-C model predictions with experimental data from Shokrieh et al. [42] for neat PP.

Despite minor deviations at the very onset of deformation, the reconstructed true stress–strain curves provided a highly reliable foundation for extracting the J-C flow stress parameters. The extraction algorithm relies on a sequential, two-step calibration approach. First, the quasi-static reference strain rate ($\dot{\varepsilon \;}=\;6.67\times 10^{-4}\;s^{-1}$) is isolated to determine the yield stress (A) and the strain hardening constants (B and n). This is achieved using a non-linear optimization routine that minimizes the residual errors between the experimental data and the analytical formulation. Following this, the strain-rate sensitivity parameter (C) is determined via linear regression. By evaluating the ratio of the dynamic flow stress to the quasi-static reference stress at a fixed true strain level of $\varepsilon_{True}\approx 0.148$ (equivalent to the ultimate engineering strain of ε=0.16), and plotting these values against the logarithmic strain rate, C is extracted directly from the slope. The success of this calibration strategy is visually demonstrated in Figure 7, which compares the experimental point clouds with the J-C model predictions across all tested strain rates for the neat PP matrix.

The predicted curves capture the rate-dependent yielding and the subsequent strain hardening evolution with remarkable accuracy. To quantitatively validate the numerical framework, the computed J-C parameters were compared against established literature values for neat PP. As summarized in Table 3, the parameters obtained in the present study (A=22.1 MPa, B=50.43 MPa, n=0.663, and C=0.0561) align closely with the benchmark data reported by Shokrieh and Joneidi [53]. The margin of error is particularly narrow for the critical yield stress (3.91%) and the strain hardening coefficient (5.13%). Al-though the relative deviation in the strain hardening exponent (n) appears numerically higher (21.88%), the overarching phenomenological fit to the experimental data remains highly consistent. Ultimately, this strong correlation validates the proposed parameter identification framework, confidently justifying its subsequent application to the novel nanocomposites investigated in this study.

### 3.2. SPH Bird-Strike Model Validation

**Figure 8:**
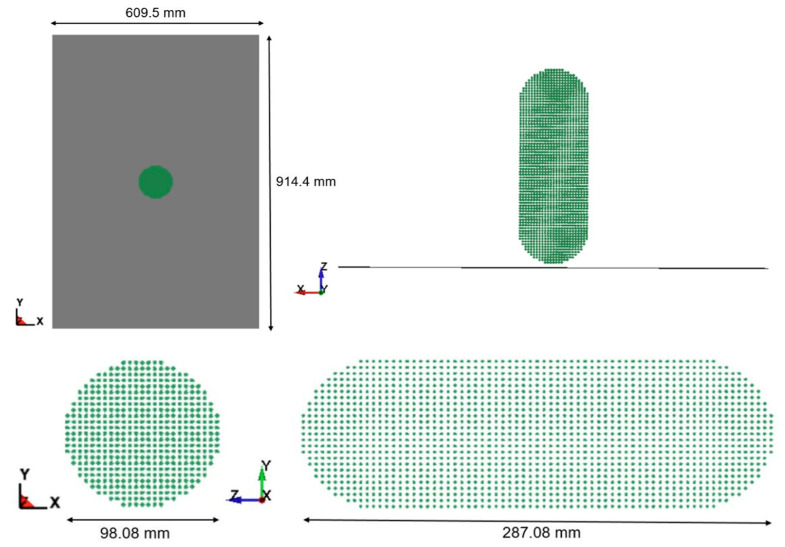
Dimensions of the target plate and SPH bird projectile employed to validate the simulation setup.

A critical aspect of this methodology was the modeling of the bird projectile; given that birds behave like fluids upon high-speed impact and undergo disintegration, traditional Lagrangian meshing often leads to excessive distortion and computational failure [66]. Consequently, the Smoothed Particle Hydrodynamics (SPH) method was adopted to simulate the bird. The bird was modeled as a cylinder with hemispherical ends, a geometry cited in the literature as yielding impact characteristics closest to real biological data with a standardized mass of 4 lb (1.81 kg) [63,84]. As detailed in Figure 8, the generated SPH bird model has an overall length of 287.08 mm and a diameter of 98.08 mm. The material behavior was defined using the MAT_009_NULL card coupled with an EoS to represent the hydrodynamic response under high pressure [63]. To ensure the reliability of the SPH parameters before applying them to the complex canopy structure, a validation study was performed against experimental data, and the simulation results were compared with those in the literature [63,84]. In this validation setup, the SPH bird model was impacted against an Aluminum 7075-T6 plate, which was modeled using the MAT_003_PLASTIC_KINEMATIC material law. The target plate and SPH bird model sizes are given in the Figure 8.

**Figure 9:**
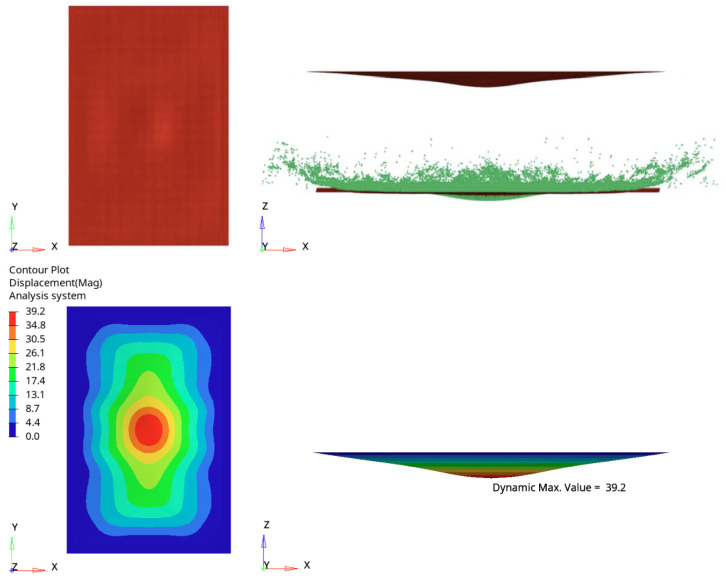
Deformation of aluminum plate (4.06 mm) impacted at 138.386 m/s.

The target plate was represented by a 2D mesh consisting of 22,326 elements and 22,632 nodes, with boundary conditions constraining the X, Y, and Z translations at the edges. Figure 9 illustrates the simulated dynamic deformation and displacement contours of a 4.06 mm thick aluminum plate subjected to an impact velocity of 138.386 m/s. The simulation effectively captures the fluidic spread of the SPH particles across the target, resulting in a localized maximum dynamic displacement of 39.2 mm at the central impact zone. This spreading pattern is a characteristic feature of high-velocity soft-body impacts, in which the dynamic pressure at the contact interface exceeds the yield strength of the biological tissue by several orders of magnitude. Under these conditions, the bird no longer behaves as a rigid or deformable solid but instead responds hydrodynamically, with its material being redirected laterally from the axial impact direction, a phenomenon commonly referred to as the ‘squash phase’ in bird-strike literature [63,84]. The SPH method is particularly well-suited to capturing this behavior, as its Lagrangian free-particle formulation naturally accommodates large deformations and material fragmentation without the mesh distortion issues that would cause conventional finite element approaches to fail.

To quantitatively assess the model’s accuracy, the simulations were expanded to evaluate varying plate thicknesses (6.35 mm, 4.06 mm, and 2.54 mm) at their respective impact velocities. The comparison between the physical test data and the current study’s numerical results was summarized in Table 4. The computational model demonstrates strong predictive capabilities, particularly for the 4.06 mm plate, which yielded a dynamic displacement of 39.2 mm, an error margin of just 3% compared to the 38.1 mm experimental baseline. Furthermore, the simulation successfully predicted complete material failure for the thinnest plate (2.54 mm) impacted at 141.472 m/s, showing good agreement with the experimental outcome. For the 6.35 mm plate, a higher deviation of approximately 20% is observed (30.4 mm predicted vs. 25.4 mm measured). This level of discrepancy is consistent with literature reports for high-velocity impact on thick plates (Furtado, [63]), and is further supported by Walvekar et al. [84], who compared SPH simulations with experimental data for similar configurations. The relatively higher error can be attributed to several factors, including the use of shell elements to model the plate, the increase in structural stiffness with thickness, SPH particle discretization, contact algorithm formulation, and simplifications in the material model. Considering these factors, the level of agreement is considered acceptable for validation of the SPH bird model across all tested plate thicknesses, particularly for the 4.06 mm and 2.54 mm plates, where lower deviations and failure predictions were observed.

### 3.3. Macro-Scale Impact Simulation Bird-Strike Results for Neat PC

Neat PC was first characterized using the Goldberg constitutive model to reproduce the experimental engineering stress–strain response over the strain-rate range relevant to the present study. The identified Goldberg parameters for the neat PC matrix were obtained by fitting the model to the experimental data reported by Sarıkaya et al. [21]. The calibrated parameters were n=1.508, $Z_{0}=40.0$ MPa, $Z_{1}=210.2$ MPa, q=144.33, $\alpha_{0}=1.5$, and $\alpha_{1}=0.0$. In addition, the elastic modulus E=2560 MPa and the reference inelastic strain-rate parameter $D_{0}=10^{6}\;s^{-1}$ were adopted directly from the literature [21,36,42]. For the present calibration, the fitting procedure was limited to 0–0.16 engineering strain. This limit was selected deliberately to maintain numerical stability, remain consistency with the preliminary PP-based validation framework, and focus the constitutive calibration on the strain regime most relevant to the subsequent bird-strike simulations.

**Figure 10:**
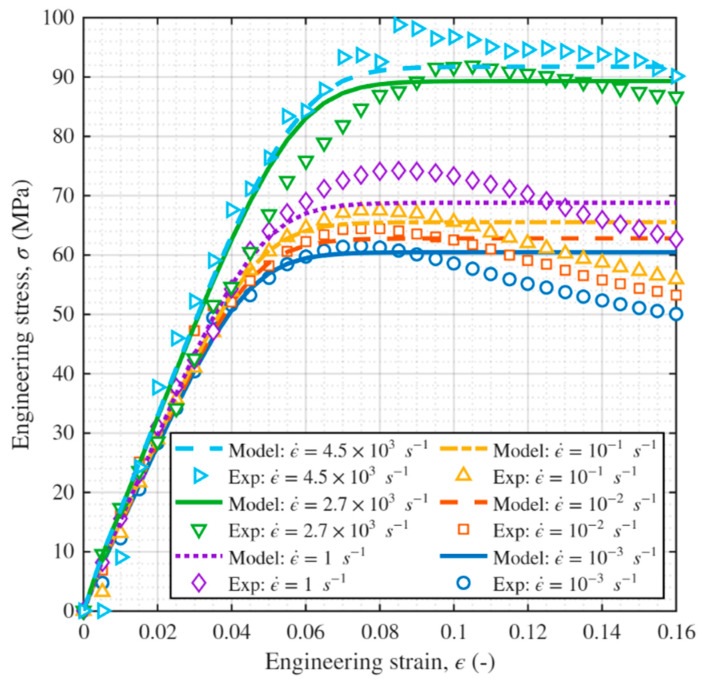
Neat PC baseline behavior using the Goldberg model.

The Goldberg formulation provides a reliable description of the pre-peak and moderate post-yield region, but it was not able to represent the full stress drop at larger strains in a physically consistent manner. As shown in Figure 10, the model captures the initial stiffness, the strain-rate-dependent increase in flow stress, and the general hardening trend with reasonable agreement.

**Figure 11:**
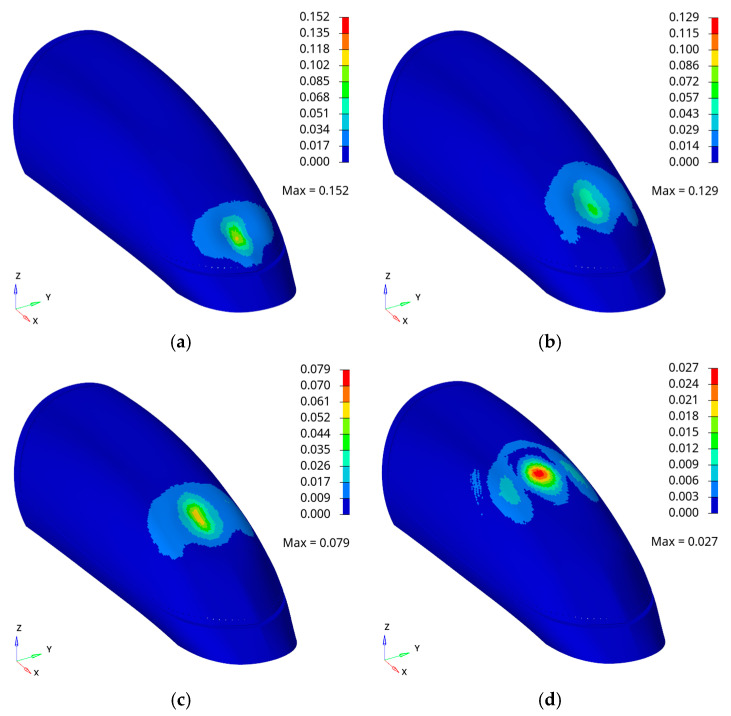
Effective strain contours of the neat PC canopy at four critical impact locations: (**a**) P1, (**b**) P2, (**c**) P3, and (**d**) P4.

To confirm the adequacy of the chosen strain limit, the effective strain contours for the baseline neat PC canopy are presented in Figure 11 for the four critical impact locations. The results clearly show that the highest strain concentration occurs in a localized zone around the impact footprint, while the surrounding canopy region experiences a more distributed but markedly lower strain level. P1 produces the highest effective strain, followed by P2 and P3, whereas P4 generates the lowest local strain demand. This choice was also supported by the strain levels observed in the bird-strike simulations, where the maximum effective strain in the critical zones reached approximately 15% at the most severe impact location; therefore, using 16% as the upper fitting bound provided a conservative margin while remaining consistent with the constitutive calibration strategy adopted in similar polymer studies.

Through the bird-strike analysis of neat PC, the baseline Johnson–Cook parameters taken from the reference study [21] were A=55 MPa, B=68 MPa, n=2.64, and C=0.037, whereas the parameters obtained from the present calibration were A=56.46 MPa, B=6.69 MPa, n=0, and C=0.040. Although the resulting hardening constants differ from the reference set, the effect of these differences on the structural bird-strike response was examined through a dedicated sensitivity study, discussed below. Accordingly, the first bird-strike simulations were carried out using the reference J-C parameters as a baseline, and the calibrated set was then evaluated to assess its influence on the predicted canopy deformation.

The maximum displacement results from the sensitivity study, which included five different cases, were evaluated at P1 and are summarized in Table 5. The comparison shows that modifying B alone does not significantly affect the maximum displacement, which remains approximately 118.6 mm in both the baseline case and Case-1. Even when B and n are changed simultaneously, the displacement decreases only moderately to 115.2 mm. A similarly limited variation is observed when B and C, or A and B, are modified together. Finally, the fully updated model yields a maximum displacement of 114.0 mm, corresponding to only a modest reduction relative to the baseline value. The J-C hardening parameters appear to have only a limited influence on the maximum displacement under the current simulation conditions. Strains developed in the canopy remain relatively low, with maximum effective strains of ~15% occurring on the inner surface of the canopy at the most critical impact location (P1). Consequently, the material response is dominated by the elastic and early plastic regime, and the strain hardening region is not fully activated. Even significant variations in the hardening parameters (B and n) therefore lead to only minor differences in predicted displacement. For higher strain levels or alternative impact conditions, where the material enters a more pronounced hardening regime, the sensitivity to these parameters is expected to increase. While this peak strain remains well below the dynamic plastic true fracture strain of PC (D1 = 0.75) reported by Sarıkaya et al. [21], which further increases at high strain rates due to adiabatic heating, it falls within the range associated with local neck formation and potential crazing initiation. Accordingly, the present simulations should be interpreted strictly as a deformation-based assessment: global rupture and perforation are not anticipated under the considered loading conditions, whereas the possibility of localized crack initiation at P1 cannot be fully excluded. Incorporation of an explicit crack-propagation criterion remains an important direction for future work.

**Figure 12:**
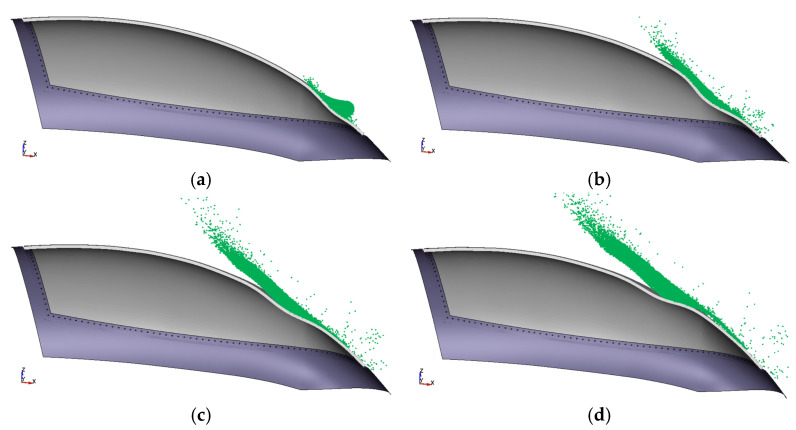
Baseline bird-strike progression at different time steps for P1: (**a**) *t* = 0.95 ms, (**b**) *t* = 2.4 ms, (**c**) *t* = 3.95 ms, and (**d**) *t* = 4.95 ms.

The baseline bird-strike sequence at P1 is illustrated in Figure 12 at four representative time instants. At the early stage of impact (t=0.95 ms), the bird first establishes contact with the canopy outer surface and the local deformation begins to develop. At this extreme impact velocity of 236 m/s, the stresses generated at the contact interface far exceed the strength of the bird’s biological tissue, causing it to behave hydrodynamically rather than as a solid projectile. As the event progresses to t=2.4 ms, the projectile spreads rapidly along the canopy due to the fluid-like behavior of the bird under high-velocity impact. At t=3.95 ms, the bird material is further dispersed over a wider contact area, while the canopy undergoes its major transient deformation. By t=4.95 ms, the bird has largely fragmented and flowed over the transparency, and the main impact phase is essentially completed. This sequence highlights the highly transient nature of the loading and confirms that the response is governed by a localized high-rate interaction followed by rapid projectile disintegration and load redistribution over the canopy surface. The progressive fragmentation of the SPH particle cloud, clearly visible across the four time steps, is a direct consequence of the hydrodynamic behavior of the bird under extreme dynamic pressure, and is accurately captured by the free-particle nature of the SPH formulation.

**Figure 13:**
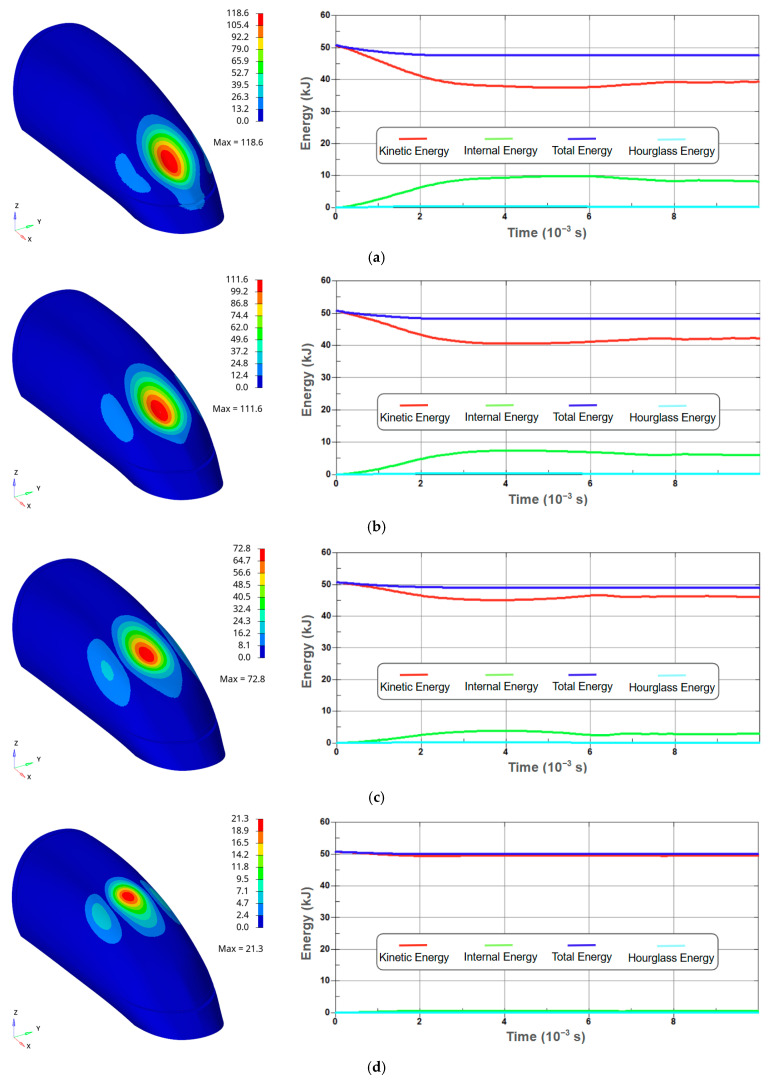
Total deformation contours of the neat PC canopy at four critical impact locations: (**a**) P1, (**b**) P2, (**c**) P3, and (**d**) P4.

After establishing the baseline model for neat PC, the maximum deformation response of the canopy at four critical impact locations was evaluated. The results indicate that the largest deformation occurs at P1, reaching approximately 118.6 mm. At positions 2, 3, and 4, the corresponding maximum deformations are 111.6 mm, 72.8 mm, and 21.3 mm, respectively, demonstrating the strain distribution along the canopy surface. These deformation contours, as illustrated in Figure 13, clearly capture the areas of highest strain concentration and provide a direct visualization of structural response under impact. Furthermore, the energy balance curves for the neat PC model provide critical insights into the underlying impact mechanics. Considering a 1.8 kg bird striking the canopy at an initial velocity of around 236 m/s (460 knots), the system’s initial kinetic energy was calculated to be around 50 kJ. As shown in the energy histories corresponding to the total deformation contours (Figure 13), all reported energy components correspond to the entire numerical system. Prior to impact, however, the kinetic energy is initially carried entirely by the bird model. Upon impact at the most severe location (P1), the kinetic energy decreases from approximately 50 kJ to 38 kJ, while the internal energy rises to nearly 10 kJ due to elastic and plastic deformation of the canopy. This behavior indicates that only partial momentum transfer occurs during impact, primarily due to the curved canopy geometry and the frictionless contact definition, which allow part of the SPH bird material to spread and slide along the canopy surface after the initial contact phase. The slightly larger reduction in total energy observed at P1, and to a lesser extent at P2, is associated with the more pronounced local deformation and SPH particle spreading occurring at these impact locations, where the curved canopy geometry promotes stronger lateral particle flow during impact. Nevertheless, throughout the event, the total energy remains effectively constant, with only minor numerical fluctuations associated with contact interactions and the explicit time integration scheme. In addition, the simulation domain was defined through a balance between minimizing SPH particle loss and maintaining computational efficiency, while remaining sufficiently large to prevent particle escape within the 10 ms simulation duration. Importantly, the artificial hourglass energy remains near zero across all four impact locations, confirming the effectiveness of the hourglass control implementation and the numerical stability of the simulation. These results validate the neat PC baseline at all four impact locations, indicating that variations in B, n, and C have limited effect on displacements. This reference provides a basis for assessing the energy absorption and structural performance of nanocomposites reinforced with silica, alumina, and zirconia under high-velocity impact conditions.

### 3.4. Macro-Scale Impact Simulation Bird-Strike Results for PC Nano Composites

**Figure 14:**
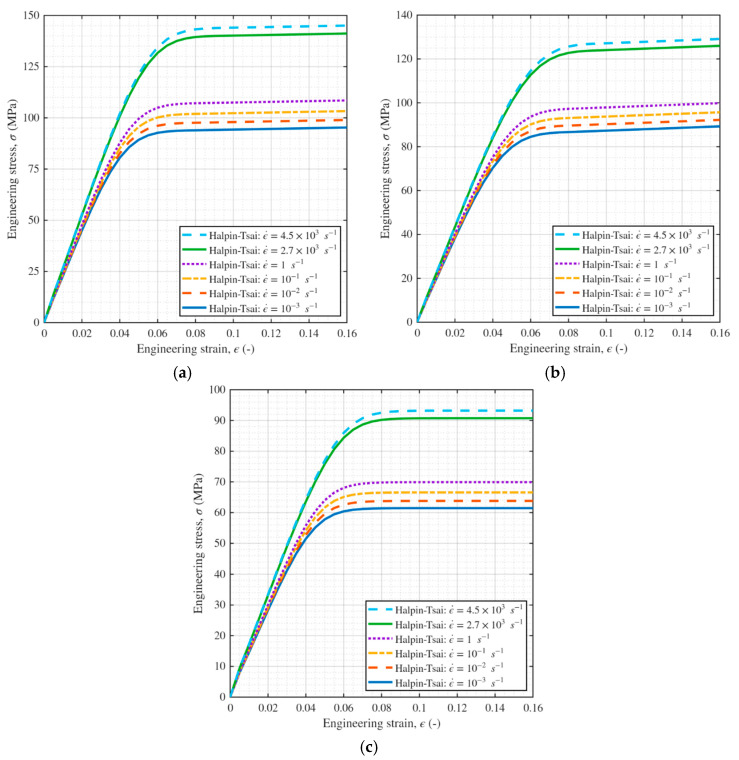
Halpin–Tsai micromechanical predictions of nanocomposite PC: (**a**) silica-reinforced PC at 30 wt%, (**b**) alumina-reinforced PC at 2 wt%, and (**c**) zirconia-reinforced PC at 2.5 wt%.

This section focuses on generating stress–strain curves for nanoparticle-reinforced PC and conducting bird-strike analyses to quantify improvements in structural deformation and to characterize changes in impact-energy absorption relative to neat PC. The SRDM approach was employed to generate dynamic stress–strain responses for each candidate nanoparticle reinforcement across a range of strain rates. The resulting curves, which serve as the foundational input for subsequent macro-scale impact simulations, are presented in Figure 14, providing a direct comparison of the strain-rate dependent mechanical behavior of the various nanocomposite formulations at their respective maximum allowable nanoparticle contents: 30 wt% for silica, 2 wt% for alumina, and 2.5 wt% for zirconia.

Figure 14 clearly shows that these materials exhibit substantial improvements in elastic modulus and yield stress, and a larger stress–strain area up to the defined strain limit, compared to neat PC. A corresponding improvement in deformation resistance is expected in the bird-strike analysis, although the effect of reinforcement on structural energy absorption must be assessed separately at the canopy level. To accurately predict these outcomes, the J-C parameters and elastic properties identified using the SRDM framework were summarized in Table 6. These parameters are crucial in modeling the rate-sensitive behavior of the materials under dynamic impact conditions, as they have been calibrated by employing the same MATLAB code as before, ensuring consistency and reliability in the analysis. Regarding the composite material properties, for the alumina- and zirconia-reinforced composites, the low nanoparticle weight fractions (≤2.5 wt%) result in negligible changes in bulk density and Poisson’s ratio relative to neat PC; therefore, these properties were assumed equal to those of the matrix. This approach is consistent with standard micromechanical modeling practices for low-volume fractions of rigid nanoparticles [80,81]. For the silica-reinforced composite, a higher nanoparticle loading (up to 30 wt%) was used, and the effective density and Poisson’s ratio were computed using a classical rule-of-mixtures approach. While the cited literature [13] does not explicitly present rule-of-mixtures calculations for PC, it experimentally validates the mechanical and optical performance of high-loading silica nanocomposites in PMMA, supporting the assumptions adopted in Table 6.

It is evident that the elastic modulus of the nanocomposites increases relative to neat PC. Notably, the modulus enhancement for the zirconia-reinforced system is smaller than that of the other two nanocomposites, despite its higher elastic modulus is than silica. This suggests that the mechanical response is governed by both the weight percentage and the nanoparticle shape factor. In the present model, zirconia is assumed to be spherical, corresponding to a constant shape factor of 2, which limits the attainable stiffness improvement. It is also important to note the validity of the proposed approach, as the elastic modulus of the alumina-reinforced nanocomposite was predicted to increase by 25% compared with the baseline material. This prediction is in close agreement with the experimental result reported by Hakimelahi et al. [81], where a 30% improvement in Young’s modulus was measured for PC reinforced with alumina whiskers at the same 2 wt% loading, as further documented in [80]. The approximately 5% difference between the SRDM prediction and the experimental benchmark is well within the acceptable bounds for micromechanical modeling, providing direct validation of the framework’s predictive accuracy for PC-based oxide nanocomposite systems.

While the nanocomposite materials exhibit superior strength under bird-strike loading, their impact performance is governed not only by strength but also by their ability to absorb and dissipate energy through deformation. Therefore, the structural response was first assessed in terms of maximum canopy deformation for all nanocomposite configurations, followed by a detailed energy-based comparison for the best-performing silica-reinforced model. It should be noted that silica reinforcement was selected for detailed analysis, as it demonstrated the most significant improvement in mechanical performance among the investigated nanofillers. The maximum deformations from the bird-strike simulations for all nanocomposite configurations are summarized in Table 7 for the four impact locations (P1–P4).

The deformation results in Table 7 show that nanoparticle reinforcement reduces the maximum canopy deformation at all four impact locations compared with neat PC. Among the investigated nanofillers, silica-reinforced PC provides the most pronounced improvement, reducing the maximum deformation at P1 from 118.6 mm to 61.9 mm, corresponding to an approximately 48% reduction. Similar reductions are also observed at P2, P3, and P4, where the maximum deformations decrease from 111.6 mm to 65.6 mm, from 72.8 mm to 39.3 mm, and from 21.3 mm to 13.1 mm, respectively. Alumina-reinforced PC also shows a clear improvement relative to neat PC, whereas zirconia-reinforced PC exhibits only a limited reduction and remains closer to the baseline response. This trend is consistent with the micromechanical predictions discussed above, where the lower allowable weight fraction and spherical particle morphology of zirconia restrict the attainable stiffness enhancement despite its high intrinsic elastic modulus. Overall, the deformation results confirm that silica reinforcement provides the most effective reduction in canopy deflection under the considered bird-strike conditions.

**Figure 15:**
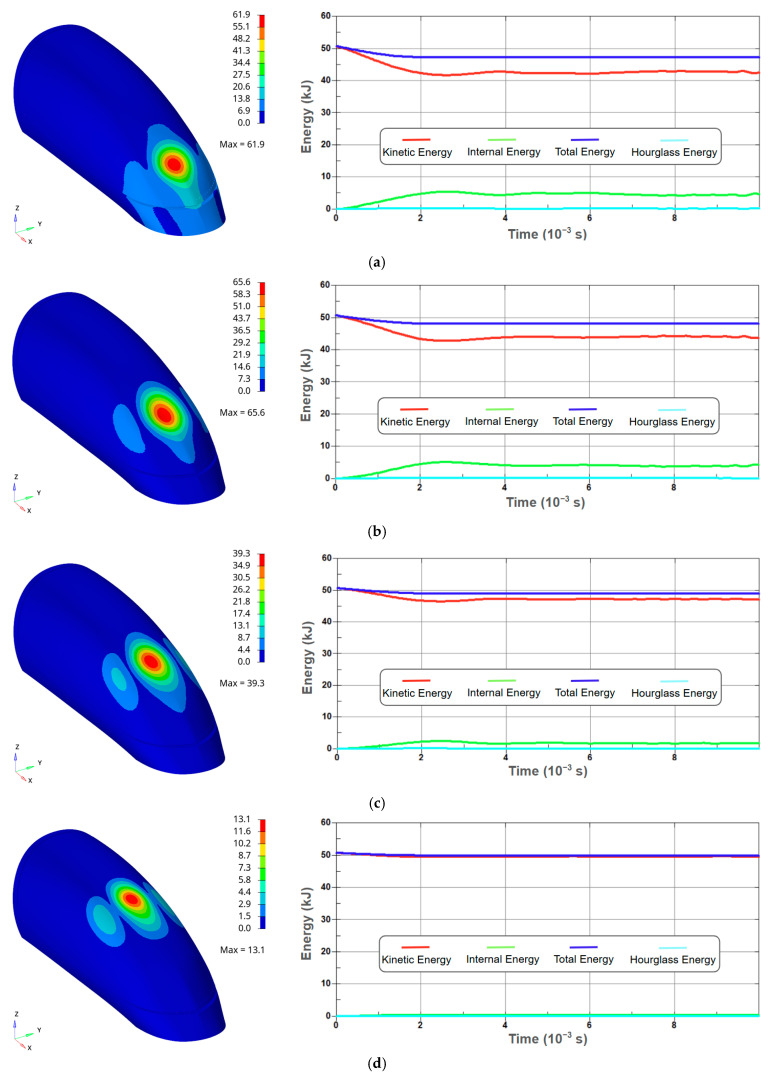
Total deformation contours of silica-reinforced PC canopy at four critical impact locations: (**a**) P1, (**b**) P2, (**c**) P3, and (**d**) P4.

Following this deformation-based comparison, the bird-strike response of the silica-reinforced PC canopy was further evaluated against the baseline model by examining the total deformation contours in Figure 15 together with the corresponding energy histories at four critical impact locations (P1–P4). Each energy component provides specific insight into the physical mechanisms governing the impact process including kinetic energy transfer, internal energy absorption, total energy conservation, and hourglass energy control.

As discussed previously for the neat PC baseline model, all reported energy components correspond to the entire numerical system, and the overall energy balance remains well conserved throughout the simulations with only minor numerical fluctuations associated with contact interactions and the explicit time integration scheme. The same numerical considerations and contact conditions are also valid for the silica-reinforced nanocomposite impact simulations presented in Figure 15.

In the present analyses, the hourglass energy remains close to zero for all impact locations and both material models, indicating that artificial deformation modes are effectively controlled and ensuring the stability and accuracy of the results. At P1, the baseline model shows a reduction in kinetic energy to approximately 38 kJ, indicating partial momentum transfer and energy dissipation through canopy deformation, whereas the silica-reinforced model retains higher kinetic energy levels (~41–42 kJ), suggesting reduced energy transfer to the structure and greater residual kinetic energy after impact; similarly to the baseline model discussed in Section 3.3, this behavior is associated with the curved canopy geometry, frictionless contact condition, and the resulting SPH particle spreading along the canopy surface during impact. Consistent with the baseline model discussed in Section 3.3, a slightly larger reduction in total energy is also observed at P1, and to a lesser extent at P2, due to the more pronounced SPH particle spreading at these impact locations. The internal energy reflects the amount of energy absorbed by the structure through deformation, including elastic and plastic strain energy, and at P1, the baseline model reaches higher internal energy levels (~10 kJ), whereas the silica-reinforced model shows a reduced value (~5 kJ), confirming that the baseline material dissipates more impact energy through deformation, while the addition of silica limits energy absorption due to increased stiffness; the more confined deformation zones observed in Figure 15 further support this interpretation, indicating reduced strain distribution in the reinforced model. At P2 and P3, the same trend persists, although the overall impact severity decreases, with the silica-reinforced model consistently exhibiting higher kinetic energy and lower internal energy compared to the baseline model, demonstrating that the stiffening effect of silica reduces structural compliance not only at the most critical location but also across intermediate regions of the canopy; the deformation contours show that energy absorption becomes increasingly localized, which may lead to higher stress concentrations despite lower overall deformation. At P4, the responses of both models become nearly identical, as the shallow impact angle causes the projectile to undergo a grazing interaction with the canopy, resulting in minimal kinetic energy loss and negligible internal energy generation; consequently, under such conditions, the impact behavior is governed primarily by contact kinematics rather than material stiffness.

Overall, the combined analysis of energy components demonstrates that nanoparticle reinforcement introduces a clear trade-off between stiffness and energy absorption. Although the addition of nanoparticles increases the elastic modulus and yield stress of the PC canopy, the internal energy is lower than that of the baseline model because the reinforced material experiences much lower strain levels and exhibits little to no plastic deformation, so the area under the stress–strain curve is not fully utilized. In this study, the maximum allowable reinforcement weight percentage was used, constrained by optical transmittance requirements; however, ideally, the reinforcement rate should be optimized to balance stiffness and energy absorption. Higher stiffness is necessary to protect the pilot and HUD equipment and to maintain structural integrity, while energy absorption is required to minimize shocks transmitted to the airframe and to achieve lower stress levels. From a design perspective, this suggests that material selection for bird-strike-resistant canopy systems must carefully balance stiffness and energy dissipation to achieve optimal performance. Among the investigated nanofillers, silica demonstrated the most balanced overall performance for transparent canopy applications. Although alumina and zirconia provided localized improvements in stiffness and impact resistance, silica reinforcement resulted in the most significant reduction in canopy deformation while also offering superior optical compatibility with the PC matrix due to its relatively close refractive index. In addition, silica nanoparticles permit comparatively higher allowable loading fractions while maintaining transparency, making them more favorable from both manufacturability and optical design perspectives. Therefore, considering the combined effects of mechanical performance, optical feasibility, and processing constraints, silica-reinforced PC emerged as the most suitable candidate within the investigated design space.

## 4. Conclusions

This study systematically investigated the mechanical response of neat and nanocomposite polycarbonate (PC) under high-strain-rate loading, with a particular focus on bird-strike impact performance in aircraft canopy applications. The SRDM-based predictions of Johnson–Cook parameters and stress–strain behavior were first validated against previously published polypropylene (PP) experimental data, demonstrating the accuracy and reliability of the modeling framework for strain-rate dependent polymers. Nanocomposite reinforcement consistently improved the elastic modulus and overall structural response relative to neat PC, with silica-reinforced PC demonstrating the most balanced overall performance in terms of bird-strike resistance, optical compatibility, and manufacturability among the investigated nanofillers. The results indicate that nanoparticle shape factor plays a critical role in determining the mechanical response: spherical zirconia particles, with a low shape factor, provided limited modulus improvement despite their high elastic modulus. This highlights that both particle geometry and weight percentage significantly influence the effectiveness of reinforcement. Bird-strike simulations revealed that reinforced canopies experience lower maximum deformations and more favorable structural response than the baseline model. Analysis of kinetic, internal, total, and hourglass energies indicated that silica-reinforced PC reduces peak deformation, but does not provide superior internal energy dissipation, compared to the neat PC. These findings underscore the inherent trade-off between increased stiffness and energy absorption, highlighting the necessity to optimize reinforcement levels to balance structural protection and impact energy dissipation. Overall, the SRDM framework demonstrates a computationally efficient and predictive approach for capturing the rate-dependent behavior of polymer nanocomposites, offering a tractable alternative to full molecular dynamics (MD) simulations. Additionally, practical considerations such as particle agglomeration and optical transparency impose limits on filler content, which must be accounted for in the design of transparent canopy materials. Despite the promising findings, several limitations of the present framework should be acknowledged. The optical screening criteria adopted in this study were based on experimentally reported transparent nanocomposite systems available in the literature. Accordingly, future work could include the development of a quantitative optical transmittance model to predict transparency under varying nanoparticle loadings and dispersion conditions, together with additional experimental and molecular dynamics validation studies on transparent PC-based nanocomposites. Furthermore, the present simulations were limited to oblique impact scenarios; near-normal impact orientations may occur during descent or maneuvering flight. Future work may therefore extend the present analyses to different impact orientations and loading conditions representative of realistic flight scenarios. Additionally, the present framework does not explicitly incorporate interfacial damage mechanisms, nanoparticle agglomeration distributions, fracture criteria, or thermal effects, which may influence the impact response under more severe loading conditions. Future work may also extend this approach to other transparent polymer systems and explore the influence of nanoparticle alignment, distribution, and multiscale effects on dynamic fracture and failure mechanisms, enabling direct comparison between SRDM-based predictions and molecular dynamics analyses. Addressing these aspects would not only improve the model’s comprehensiveness but also support the design of next-generation transparent nanocomposite canopy systems.

## Figures and Tables

**Table 1:** Mesh-convergence results for the extracted polycarbonate canopy submodel at impact P2 using different average element sizes.

Average Element Size (mm)	Total Number of Elements	Maximum Displacement (mm)	Relative Change (%)
30	2340	22.8	-
20	5148	54.7	139.9
15	9360	73.5	34.4
10	21,060	82.3	12.0
5	83,304	84.4	2.6

**Table 2:** Mechanical, physical, and optical properties of selected nanoparticles considered for reinforcing the PC matrix.

Nano Particles	Type	Refractive Index (*n*)	Young’s Modulus (E) GPa	Poisson’s Ratio (ν)	Density (g/cm^3^)	Size (nm)	Max %wt Allowance
Silica (SiO_2_) [13]	Spherical	1.45–1.46	~72	0.17	2.2	Ø: 10–40	30
Alumina (Al_2_O_3_) [31,81,82]	Nanowhisker	~1.76	~530	0.21	3.86	Ø: 2–4 L: 2800	2
Zirconia (ZrO_2_) [72,83]	Spherical	~2.16	~200	~0.30	5.68	Ø: 5	2.5

**Table 3:** Comparison of the present and literature J-C parameters for neat PP.

Data Type	A (MPa)	B (MPa)	n	C
Shokrieh and Joneidi [53]	23	47.97	0.544	0.0643
Present Study	22.1	50.43	0.663	0.0561
Error Margin (%)	3.91	5.13	21.88	12.75

**Table 4:** The comparison between experiment and simulation result [63].

Plate Thickness	Impact Velocity	Displacement Test Result	Displacement Study Result	Error Margin (%)
6.35 mm	136.328 m/s	25.4 mm	30.4 mm	20
4.06 mm	138.386 m/s	38.1 mm	39.2 mm	3
2.54 mm	141.472 m/s	Material Failure	Material Failure	-

**Table 5:** Sensitivity of maximum displacement to J-C parameters for neat PC at P1.

Cases	Change	A (MPa)	B (MPa)	n	C	Max. Displacement (mm)
Baseline [21]	-	55	68	2.64	0.037	118.6
Case-1	B	55	6.69	2.64	0.037	118.6
Case-2	B and n	55	6.69	0	0.037	115.2
Case-3	B and C	55	6.69	2.64	0.040	117.8
Case-4	A and B	56.46	6.69	2.64	0.037	117.8
Case-5	Model	56.46	6.69	0	0.040	114.0

**Table 6:** Johnson–Cook (J-C) parameters and elastic properties of nanocomposites PC systems reinforced with silica (30 wt%), alumina (2 wt%), and zirconia (2.5 wt%).

Nanocomposite	A (MPa)	B (MPa)	n	C	E (GPa)	Poisson’s Ratio (ν)	Density (g/cm^3^)
Silica PC (SiO_2_)	79.98	87.05	0.43	0.036	4.19	0.377	1.39
Alumina PC (Al_2_O_3_)	81.49	99.65	0.63	0.031	3.21	0.39	1.2
Zirconia PC (ZrO_2_)	63.39	144.82	1.27	0.035	2.63	0.39	1.2

**Table 7:** Maximum deformations of neat and nanocomposite polycarbonate (PC).

Material	P1 (mm)	P2 (mm)	P3 (mm)	P4 (mm)
Neat PC	118.6	111.6	72.8	21.3
Silica (SiO_2_)	61.9	65.6	39.3	13.1
Alumina (Al_2_O_3_)	89.3	85.9	53.4	17.1
Zirconia (ZrO_2_)	110.4	103.9	66.9	20.9

## Data Availability

The data used to support the findings of this study are available from the corresponding author upon request.
